# Transcriptomic Analysis of the Early Strobilar Development of *Echinococcus granulosus*

**DOI:** 10.3390/pathogens9060465

**Published:** 2020-06-12

**Authors:** João Antonio Debarba, Martín Pablo Cancela Sehabiague, Karina Mariante Monteiro, Alexandra Lehmkuhl Gerber, Ana Tereza Ribeiro Vasconcelos, Henrique Bunselmeyer Ferreira, Arnaldo Zaha

**Affiliations:** 1 Programa de Pós-Graduação em Biologia Celular e Molecular, Universidade Federal do Rio Grande do Sul, Porto Alegre 91501-970, Brazil; joaoatd@gmail.com (J.A.D.); martin@cbiot.ufrgs.br (M.P.C.S.); karina@cbiot.ufrgs.br (K.M.M.); henrique@cbiot.ufrgs.br (H.B.F.); 2 Laboratório de Biologia Molecular de Cestódeos, Centro de Biotecnologia, Universidade Federal do Rio Grande do Sul, Porto Alegre 91501-970, Brazil; 3 Laboratório Nacional de Computação Científica, Petrópolis, Rio de Janeiro 25651-075, Brazil; alegerber@lncc.br (A.L.G.); atrv@lncc.br (A.T.R.V.)

**Keywords:** *Echinococcus granulosus*, RNA-seq, transcriptome, strobilar development

## Abstract

*Echinococcus granulosus* has a complex life cycle involving two mammalian hosts. The transition from one host to another is accompanied by changes in gene expression, and the transcriptional events that underlie this transition have not yet been fully characterized. In this study, RNA-seq was used to compare the transcription profiles of samples from *E. granulosus* protoscoleces induced in vitro to strobilar development at three time points. We identified 818 differentially expressed genes, which were divided into eight expression clusters formed over the entire 24 h period. An enrichment of gene transcripts with molecular functions of signal transduction, enzymes, and protein modifications was observed upon induction and developmental progression. This transcriptomic study provides insights for understanding the complex life cycle of *E. granulosus* and contributes for searching for the key genes correlating with the strobilar development, which can be used to identify potential candidates for the development of anthelmintic drugs.

## 1. Introduction

Echinococcosis is a zoonotic parasitic infection caused by tapeworms of the genus *Echinococcus* and is considered as one of the 17 neglected tropical diseases prioritized by the World Health Organization [1,2]. The two most important forms of the disease are cystic echinococcosis (hydatidosis) and alveolar echinococcosis, caused by infection with *Echinococcus granulosus* and *Echinococcus multilocularis*, respectively. *Echinococcus* spp. have two-host life cycles with the larval stage growing in the tissues of an intermediate host (among a large variety of noncarnivorous species) and the adult stage living in the intestine of a definitive host (among few carnivore species) [3,4,5].

The *E. granulosus* larva, also called metacestode, is a fluid-filled vesicular cyst (the hydatid cyst) containing many infectious protoscoleces, the preadult forms of the parasite. In vitro studies demonstrated that protoscoleces have the unusual ability to differentiate in two different directions depending on the environmental stimulus. In the intermediate host, upon hydatid cyst rupture, protoscoleces released in the body cavity redifferentiate in a cystic direction, forming secondary hydatid cysts. In contrast, protoscoleces ingested by a carnivore, such as a dog, and exposed to the gut environment, differentiate into fully strobilated and sexually differentiated adult tapeworms [4,6,7,8].

Strobilar development is directly influenced by the host–parasite relationships and configure a key point for the parasite’s life cycle. Within the hydatid cyst, a protoscolex remains quiescent and invaginated. It only undergoes strobilation upon ingestion by a definitive host and exposure to the correct stimuli [8,9]. The nature of these stimuli is not fully known, but it is believed that chewing and proteolytic enzymes such as pepsin play considerable roles in differentiation induction, as well as temperature and the presence of bile salts. In addition, histological studies have already demonstrated that parasite contact or attachment to a substrate similar to that found on the surface of the canine gut also constitutes an important stimulus for strobilation [10,11,12]. These observations resulted in the elaboration of strategies for in vitro culture of *Echinococcus* protoscoleces in order to provide the necessary physiological conditions for parasite strobilar development [13].

Genome comparative studies carried out for *E. granulosus* [14,15,16] and *E. multilocularis* [15,17] provided evidence of considerable losses and gains of genes that may be associated with adaptations to parasitism. The 1149 megabase genome of *E. granulosus* comprises nine chromosomes, of which 10,231 genes have been identified [15]. Among them, however, crucial genes or even entire pathways of de novo synthesis of fatty acids, cholesterol, pyrimidines, purines, and most amino acids are absent. Thus, *E. granulosus* relies on the host for obtaining these nutrients. Regarding strobilar development, transcriptomic and proteomic studies have identified differentially expressed genes or proteins between larval and strobilated (adult) parasite life stages, either in the model cestode species *Mesocestodes corti* or in *E. granulosus* [18,19,20,21]. Specifically for *E. granulosus*, it was shown that bile acids have a crucial role in the differentiation of protoscoleces into adult worms [13], involving the expression of parasite bile acid receptors and transporters to stimulate the corresponding developmental pathways [15]. However, given the complexity of the strobilation process, many more molecular events and pathways are likely to play roles in the gradual morphological changes observed during the strobilar development of *E. granulosus*, but they remain essentially unknown.

In an attempt to find genes and provide evidence of molecular pathways involved with the gradual phenotypic changes triggered by strobilation stimuli in *E. granulosus*, we report here the transcriptomic profiling of the first 24 h after protoscolex in vitro induction to adult development. We performed a comparative analysis of the protoscolex transcriptomes of three time points within this 24 h window after strobilar induction, and provided an overview of early molecular events in strobilation. Our data reinforce the foundation required to elucidate the strobilar development in the context of host–parasite relationships, as well to improve new control strategies for echinococcosis and other cestodiases.

## 2. Results

### 2.1. Summary of the RNA Sequencing Data

The RNA extracted from *E. granulosus* protoscoleces collected at different time points after induction to strobilar development (Figure S1) was subjected to paired-end RNA-seq using Illumina technology. The analyzed samples comprise protoscoleces washed with phosphate-buffered saline (PBS), used as noninduced controls (Sample 1—PBS), protoscoleces washed with PBS and treated with pepsin (Sample 2—PEP), protoscoleces washed with PBS, treated with pepsin, and cultured in biphasic medium for 12 h (Sample 3—12 h) or 24 h (Sample 4—24 h). The RNA-seq resulted in a total of 30,821,916 reads for the four samples studied. The overall raw read mean quality score was high, with 98.4% of bases above Q30. After quality filtering, 30.8 million of paired-end reads (99.5%) were obtained, and 71.7% of the reads were mapped to the *E. granulosus* genome with known gene annotations. Table 1 shows the summary of the sequencing results.

### 2.2. Identification and Analyses of Transcripts Detected in the Transcriptome

#### 2.2.1. Number of Identified Genes

**Figure 1:**
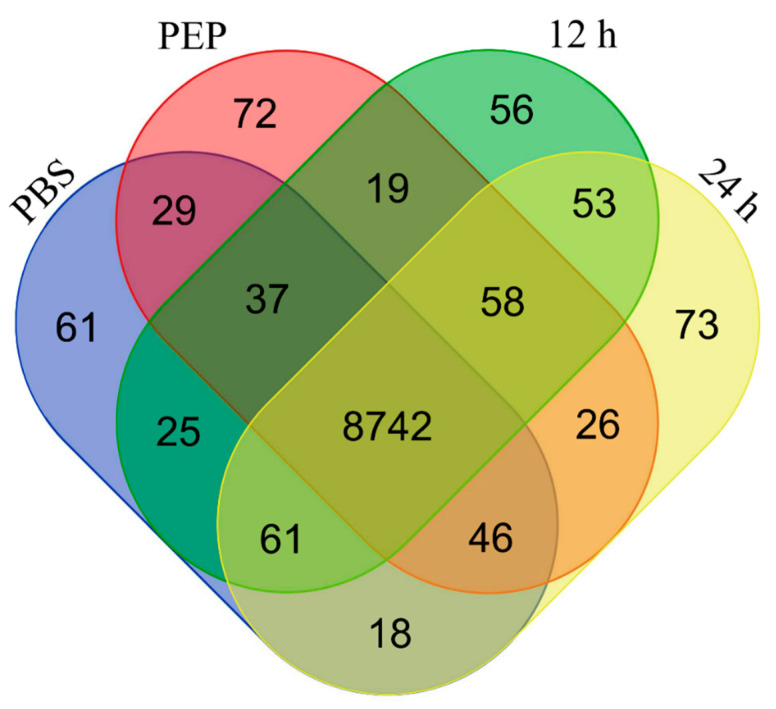
Venn diagram showing the distribution of genes between *E. granulosus* protoscolex samples. Genes with nonzero reads per kilobase million (RPKM) are represented and compared to show the number of genes with overlapping expression in four samples analyzed.

A total of 9376 different genes were represent in our dataset. A total of 9019, 9029, 9051, and 9077 genes were found in PBS, PEP, 12 h, and 24 h samples, respectively. Most of the genes (8742 genes) were represented in the four analyzed samples, but 61, 72, 56, and 73 genes were exclusively detected in PBS, PEP, 12 h, and 24 h samples, respectively (Figure 1). A complete list of the identified genes is provided in Table S1.

**Figure 2:**
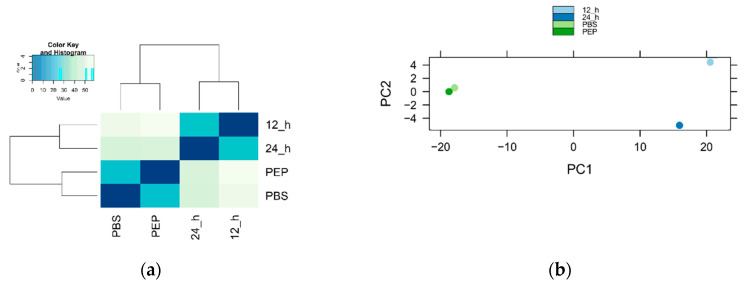
Correlation analysis of sequenced samples. (**a**) Pearson correlation coefficients between samples; (**b**) principal component analysis.

The variation between samples was calculated with Pearson correlation and principal component analysis (PCA). As shown in Figure 2, PBS and PEP samples showed higher correlation coefficients than the 12 h and 24 h samples, indicating that there was little variation among them and a separation from the other samples.

#### 2.2.2. Differentially Expressed Genes

**Figure 3:**
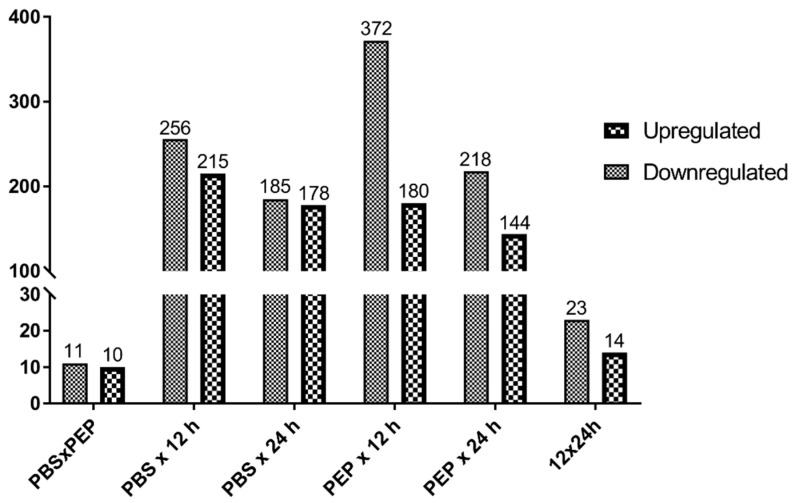
Genes differentially expressed between protoscolex samples. Up- and downregulated genes in each pairwise comparison are shown.

We performed a pairwise comparison across different samples using GFOLD. A total of 818 genes were differentially expressed between any two samples (Table S2). The number of differentially expressed (DE) genes (up- and downregulated) in each 2 × 2 sample comparison is shown in Figure 3. A predominance of downregulated genes was detected in all pairwise comparisons. PEP vs. 12 h showed the highest amount of DE genes (552 genes; 180 upregulated and 372 downregulated). Some important genes for host–parasite relationship and development are shown in Table S3, including genes such as dynein light chain, annexins, ankyrin, and tetraspanin.

**Figure 4:**
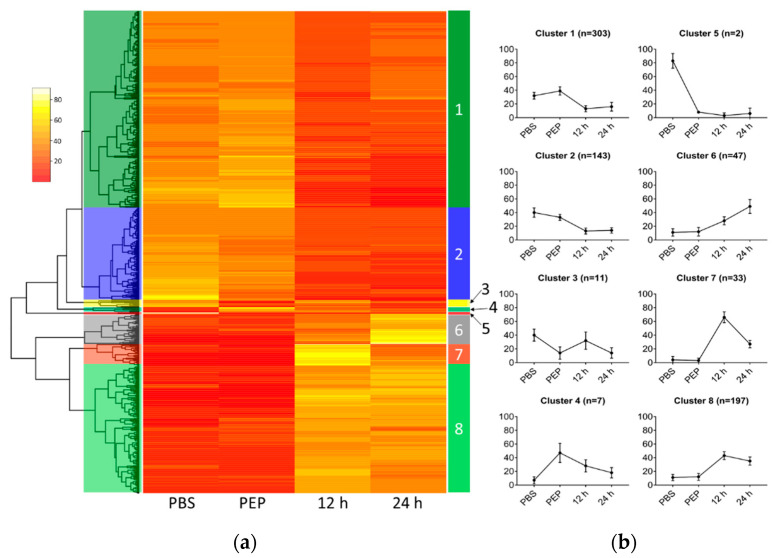
Heatmap plot of differentially expressed (DE) genes upon *E. granulosus* strobilar development induction. (**a**) Relative expression of each gene is represented in the rows and categorized clusters; (**b**) Average expression (± standard deviation) for genes in each cluster reveals similar expression patterns.

To better understand the dynamics of *E. granulosus* protoscolex gene expression upon strobilation induction, DE genes were clustered to search for similar patterns of expression between samples (Figure 4). Using the sum of the RPKM values in the four samples (cutoff > 10), 744 DE genes were categorized into eight different clusters on account of their relative expression pattern (Table S2). We computed a relative expression for each gene by dividing its expression at each time point by the sum of gene expression for all time points. In this analysis, clusters 1 and 2, including 446 genes, showed decreasing expression profile patterns throughout the treatment for protoscolex strobilation, while 277 genes in clusters 6, 7, and 8 showed increasing expression profiling patterns.

**Figure 5:**
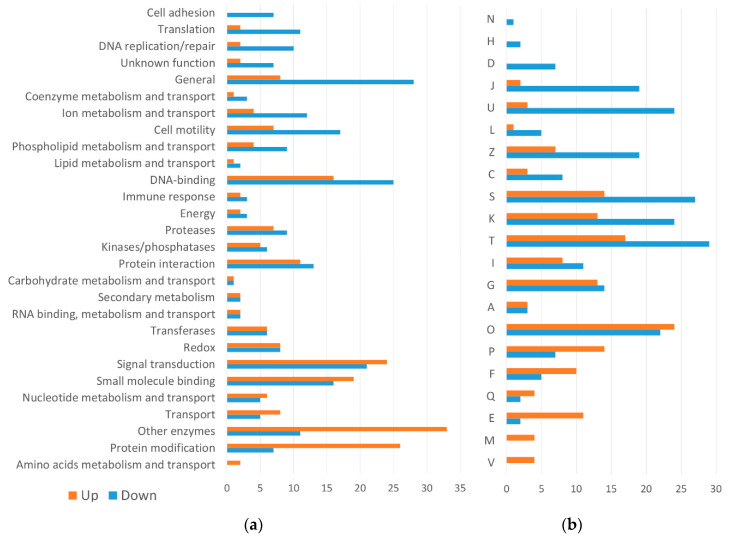
Comparative structural and functional analysis of upregulated (clusters 6, 7, and 8) and downregulated (clusters 1 and 2) DE genes. (**a**) SUPERFAMILY predictions of the conserved domains shown by percentage; (**b**) EggNOG functional categories of DE genes shown by percentage. (A) RNA processing and modification; (C) Energy production and conversion; (D) Cell cycle control, cell division, chromosome partitioning; (E) Amino acid transport and metabolism; (F) Nucleotide transport and metabolism; (G) Carbohydrate transport and metabolism; (H) Coenzyme transport and metabolism; (I) Lipid transport and metabolism; (J) Translation, ribosomal structure and biogenesis; (K) Transcription; (L) Replication, recombination, and repair; (M) Cell wall/membrane/envelope biogenesis; (N) Cell motility; (O) Post-translational modification, protein turnover, and chaperones; (P) Inorganic ion transport and metabolism; (Q) Secondary metabolites biosynthesis, transport, and catabolism; (S) Function unknown; (T) Signal transduction mechanisms; (U) Intracellular trafficking, secretion, and vesicular transport; (V) Defense mechanisms; (Z) Cytoskeleton.

A structural and functional annotation of the DE genes is summarized in Figure 5 and Table S2. The most representative domains found among the genes downregulated during early worm development (cluster 1 and 2) were related to “cell motility”, “cell adhesion”, “DNA-binding”, “translation”, and “DNA replication/repair”. Among the genes upregulated during early worm development (cluster 6, 7, and 8), the most representative domains were “signal transduction”, “other enzymes”, and “protein modification”. By EggNOG analysis, the most significant functions found in downregulated genes were “translation, ribosomal structure, and biogenesis”, “intracellular trafficking, secretion, and vesicular transport”, and “cytoskeleton”. Among the upregulated genes, the most relevant functions were “inorganic ion transport and metabolism”, “nucleotide transport and metabolism”, and “amino acid transport and metabolism”.

Uncharacterized proteins represent 270 of total DE genes, with 68 annotated as expressed conserved proteins, 62 expressed proteins and 140 hypothetical proteins (Table S2). Of these, only 10 have conserved domains predicted by SUPERFAMILY and 13 have EggNOG functional categories (three of them present in both classifications).

## 3. Discussion

Important morphological and biochemical changes occur throughout the life cycle of parasitic organisms and are probably the result of regulated changes in gene expression in response to environmental stimuli, such as host change, and temperature and pH shifts [22,23,24]. These regulated responses contribute to the mechanisms related to the development of the parasite, including strobilation and sexual maturation, as well as the evasion of the host’s immune response.

Based on the Jacob–Monod model, a hypothetical but logical model was proposed to explain how the gene expression regulation can be involved in *Echinococcus* development [8,25]. Although both the morphological characteristics of the strobilar development and the genome of *E. granulosus* are known, the correlation between these two pieces of information and the model previously proposed is still poorly understood. In this work, we provided a transcriptional analysis of *E. granulosus* protoscoleces in vitro induced to strobilar development in attempt to find genes involved in this process.

The strobilar development in *E. granulosus*, as well as other cestodes, allows adult parasite adult to have larger numbers of reproductive organs, allowing an increase in fertility [26] and makes this process an important target for study. Based on the classic works of Smyth and collaborators [13], we have previously reported an in vitro culture of *E. granulosus* protoscolex strobilar development based on a biphasic medium containing the bile salt taurocholate [21]. In this work, we cultivated protoscoleces in biphasic medium for 12 or 24 h. Here, we subjected *E. granulosus* protoscoleces to induction of strobilar development and analyzed gene expression of parasite samples collected at different time points of this process, including untreated protoscoleces washed with PBS, protoscoleces treated with pepsin, and protoscoleces cultivated in biphasic medium for 12 or 24 h. By sample-to-sample correlation analysis, it was possible to observe that the most of the identified transcripts were shared between the different samples analyzed. PBS and PEP samples have a relatively high correlation coefficient. However, with the subsequent activation of the protoscoleces and cultivation biphasic medium, mimicking the developmental transition in the definitive host, a change in the identity of genes was observed.

We identified transcripts corresponding to 9376 genes (91.6%). This number represents almost 1000 genes more than the 8393 genes identified in a previous RNA-seq of protoscoleces extracted from a single porcine liver cyst [15]. Another transcriptomic analysis found 7471, 6976, 3811, and 7724 genes in the protoscoleces, cyst germinal cells and membranes, adult worms, and oncospheres, respectively [16]. Some of these genes probably are stage-specific and transiently expressed during the parasite life cycle, indicating a possible role in adaptation and strobilation of the parasite.

Among the most expressed transcripts found in our data, it was verified a remarkable presence of those corresponding to fatty acid binding protein (FABP) and the antigen B genes. These genes have already been described among those most highly expressed *Echinococcus* genes [15,27]. The importance of these genes lies in the fact that cestodes are unable to synthesize fatty acids and cholesterol de novo [15]. They depend essentially on the sequestration, uptake, and utilization of host lipids by proteins such as FABP and antigen B. In this study, FABP genes are downregulated during *E. granulosus* adult worm development, although it has already been described that egfabp2 (EgrG_000549800) is mainly transcribed in adult and cyst [16,28]. Antigen B has both up- and downregulated genes coding its subunits, in agreement with previous works [29,30]. This variation in the composition of antigen B may be related to different properties of each subunit in binding to lipids and evasion of the immune response.

Among genes downregulated during protoscolex strobilar development, we found several genes coding for dynein light chain, oxalate:formate antiporter, and annexins. Dynein is a family of cytoskeletal motor proteins involved in intracellular motility of vesicles and organelles along microtubules and are associated with transforming growth factor (TGF)-β signaling [15,31]. Previous studies showed the expansion of this family in *E. granulosus* and schistosomes when compared to nematodes [14,15]. Oxalate:formate antiporter is a subfamily of the major facilitator transporter family, responsible for the transport of small solutes [32], but its function is not fully understood in parasites. Annexins, in contrast, are considered to play critical roles in parasite process related to the maintenance of cell integrity and modulation of the host immune responses [33]. A similar pattern of expression was observed in *M. corti*, where several genes encoding annexins were more expressed in the tetrathyridium (larval) than in the strobilated worm stage [19]. Therefore, the decrease in the expression of the annexins may be related to the absence of contact with the host in the in vitro cultures.

On the other hand, we found ankyrin, tetraspanin, heat shock protein 70 (Hsp70), and sodium bile acid cotransporter among the upregulated DE genes. Ankyrins are involved in functions such as cell cycle regulation, transcriptional regulation, cytoskeleton interactions, signal transduction, development, and intracellular trafficking [34,35]. In parasites, tetraspanins are involved in the coordination of signal transduction, cell proliferation, adhesion, migration, cell fusion, and host–parasite interactions [36]. In *E. granulosus*, tetraspanins were mostly present in the tegument and could contribute to the parasite nutrition and differentiation [37,38]. Therefore, the result of coordinated functions performed by ankyrins and tetraspanins (upregulated) in contrast to dynein light chain and annexins (downregulated) can be important to cytoskeletal remodeling associated with evagination and elongation of protoscoleces. The Hsp70s are part of the group of the expanded domain families in *E. granulosus* and may have important roles in protein folding and in protecting cells from stress [15]. The expression of several Hsp70 genes may be particularly associated with the stressful conditions of strobilation induction, which involves an increase in protein synthesis [21,39]. In turn, sodium bile acid cotransporter is an integral membrane glycoprotein that, in humans, participate in the enterohepatic circulation of bile acids. Bile acids seem to play a key role in the differentiation of *Echinococcus* protoscoleces into adult worms, and the expression of bile acid receptors and transporters may be stimulated during strobilar development [13,16].

When we analyzed the molecular function of DE genes, we also found differences between clusters. In cluster 1, which presents a downregulated expression pattern, we observed the presence of more basal functions, such as translation (e.g., Eukaryotic translation initiation factor 5a), DNA replication, and cell motility. In contrast, the upregulated DE genes of cluster 8 are related to specialized functions such as signal transduction (e.g., tyrosine protein kinase and G protein coupled receptor), enzymes (e.g., hexokinase and phospholipase), and protein modifications (e.g., Hsp70), which might correlate with the increased morphological complexity of the adult tapeworm compared to the metacestode.

In our previous work, we identified proteins newly synthesized by *E. granulosus* protoscoleces upon the induction of strobilar development [21]. Although the samples analyzed by the proteomic approach and in the present transcriptomic analysis do not correspond to the same strobilar development stages, some results are similar between these two studies. We observed a predominance of upregulated genes/proteins related to the cytoskeleton, energy metabolism, and cellular communication functions. Specifically, the 2-amino-3-ketobutyrate coenzyme A ligase, whose transcript was identified here as upregulated during strobilar development, was previously identified as a protein synthesized in response to strobilation stimuli.

Our group has recently compared the genome content of 10 parasitic platyhelminth species with the aim of identify genes and proteins related to the strobilation process [40]. Among the genes that were considered strobilation-related with unknown function, three are differentially expressed in the transcriptome here analyzed. We find that EgrG_000105500 is upregulated in *E. granulosus*, in the same way as observed in *E. multilocularis* [15] and *M. corti* [19], in the comparison between larval (pre-strobilated) and adult (strobilated) stages. EgrG_000518800 (upregulated) and EgrG_000701500 (downregulated) also show similarity to the pattern observed in *E. multilocularis* [15]. It is important to note that a large number of genes (270 DE genes—33.0%; 2976 in total—31.7%) have so far not been characterized, which makes more accurate analyses difficult, and highlights the extent to which strobilation and other *Echinococcus* developmental processes are still poorly known.

## 4. Materials and Methods

### 4.1. Parasite Material and In Vitro Cultivation

*E. granulosus* protoscoleces (G1 genotype) were aseptically collected from one hydatid cyst recovered from a naturally infected liver of cattle routinely slaughtered at a commercial abattoir (São Leopoldo, RS, Brazil). The viability of protoscoleces was determined by trypan blue exclusion test and confirmed based on their motility characteristics under light microscopy [41]. Protoscoleces were washed three times with PBS, pH 7.4 and genotyped by one-step PCR and RFLP [42]. PSCs were cultured as previously described [21]. Briefly, after washing in PBS, they were used as noninduced controls (Sample 1—PBS), or treated for strobilation induction as follows. Protoscoleces were incubated for 15 min with pepsin (2 mg/mL), pH 2.0 (Sample 2—PEP), washed with PBS and transferred to a biphasic medium contained taurocholate for 12 (Sample 3—12 h) or 24 h (Sample 4—24 h). Protoscoleces in the control and induced conditions were further maintained in culture to confirm the characteristic morphological changes of early strobilar development.

### 4.2. RNA Extraction

Total RNA from each parasite sample was extracted using TRIzol reagent, according to the manufacturer’s instructions, followed by treatment with RNase-free DNase I (Sigma, St. Louis, MO, USA) to remove DNA contaminants. The integrity of the extracted RNA was monitored using gel electrophoresis on a 1% agarose gel. RNA concentration was determined using Qubit (Thermo Fisher Scientific, Waltham, MA, USA).

### 4.3. cDNA Library Construction and Sequencing

For cDNA libraries, 4 μg of total RNA were used as start material. PBS, PEP, 12 h, and 24 h sample libraries were constructed, without replication, using the TruSeq Stranded mRNA LT Sample Preparation Kit (Illumina, San Diego, CA, USA) according to manufacturer’s instructions. Library quality control was performed using the 2100 Bioanalyzer System with the Agilent High Sensitivity DNA Kit (Agilent, Santa Clara, CA, USA). The libraries were individually quantified via qPCR using a KAPA Library Quantification Kits for Illumina platforms (KAPA Biosystems, Wilmington, NC, USA). They were pooled together in equal amounts and sequenced in a MiSeq Sequencing System (Illumina). Paired-end reads (2 × 75 bp) were obtained using a MiSeq Reagents Kit v3 (150 cycles.)

### 4.4. Data Analysis

FastQC v0.11.2 [43] was used for checking data set quality. Individual Illumina read files (fastq) were trimmed and filtered using Trimmomatic v0.36 [44]. Paired end Trimmomatic parameters used were: LEADING:10 TRAILING:10 SLIDINGWINDOW:30:20 MINLEN:30. Filtered reads were mapped to *E. granulosus* genome by using TopHat2 v2.1.0 [45] with default parameters. The genome of *E. granulosus* (PRJEB121, [15]) and annotation (version 2014-05) were retrieved from WormBase ParaSite database [46].

The reads mapped to each transcript were used to calculate normalized transcript abundance and to perform differential gene expression analysis in GFOLD v1.1.4 [47], a software package specifically designed for unreplicated RNA-seq data. Genes with |GFOLD value| > 1 or |log2 (fold change)| > 2 were considered to be differentially expressed.

Hierarchical cluster analysis and Pearson correlation coefficient were performed using the R Stats Package v3.4.0, corrplot package v0.77 and RStudio v1.0.143. The conserved functional domain structures (SUPERFAMILY, [48]) of the identified differentially expressed genes were predicted using InterProScan 5.21–60.0 [49]. The eggNOG database v4.5.1 [50] was used to acquire the functional annotation for the differentially expressed genes.

## 5. Conclusions

In this study, we have conducted RNA-Seq analyses of *E. granulosus* protoscoleces in the first 24 h of strobilar development. More importantly, this work provides information about differentially expressed genes and key molecular events activated upon *E. granulosus* strobilar development. In summary, we provide significant data that can be used to explore basic questions on the biology and evolution of cestodes, including the study of development and the host–parasite relationship. In addition, these data can be used for new echinococcosis control strategies, as well as other helminthiases.

## Figures and Tables

**Table 1:** Overview of the sequencing reads.

Samples	Q30 ^1^	Raw Reads	Filtered Reads	Mapped Reads
PBS	0.984	6,994,764	6,965,344 (99.6%)	4,909,354 (70.5%)
PEP	0.983	7,348,062	7,309,726 (99.5%)	5,090,978 (69.6%)
12 h	0.985	8,308,408	8,271,920 (99.6%)	6,103,570 (73.8%)
24 h	0.982	8,315,180	8,274,926 (99.5%)	6,085,915 (73.5%)

^1^ Q30: Phred Quality Score; probability of incorrect base call: 1 in 1000. PBS: protoscoleces washed with PBS; PEP: protoscoleces washed with PBS + treatment with pepsin; 12 h: protoscoleces subjected to PBS + PEP + biphasic medium for 12 h; 24 h: protoscoleces subjected to PBS + PEP + biphasic medium for 24 h.

## Supplementary Materials

All sequence data (raw Illumina reads) are available on the NCBI Sequence Read Archive (SRA) database under the accession ID SRP131874. The following are available online at https://www.mdpi.com/2076-0817/9/6/465/s1, Table S1: Complete list of the identified genes, Table S2: Structural and functional annotation of the differentially expressed genes, Table S3: Selected differentially expressed genes among the samples analyzed of *E. granulosus*. Figure S1: Induction of strobilar development in *E. granulosus* protoscoleces.

## References

[1] da Silva A.M. (2010). Human echinococcosis: A neglected disease. Gastroenterol. Res. Pract..

[2] WHO (2015). Investing to Overcome the Global Impact of Neglected Tropical Diseases.

[3] Eckert J., Deplazes P. (2004). Biological, Epidemiological, and Clinical Aspects of Echinococcosis, a Zoonosis of Increasing Concern. Clin. Microbiol. Rev..

[4] Thompson R.C.A., Jenkins D.J. (2014). Echinococcus as a model system: Biology and epidemiology. Int. J. Parasitol..

[5] Casulli A., Siles-Lucas M., Tamarozzi F. (2019). Echinococcus granulosus sensu lato. Trends Parasitol..

[6] Cucher M., Prada L., Mourglia-Ettlin G., Dematteis S., Camicia F., Asurmendi S., Rosenzvit M. (2011). Identification of Echinococcus granulosus microRNAs and their expression in different life cycle stages and parasite genotypes. Int. J. Parasitol..

[7] Thompson R.C.A. (1995). Biology and systematics of echinococcus. Echinococcus and Hydatid Disease.

[8] Smyth J.D. (1969). Parasites as biological models. Parasitology.

[9] Smyth J.D., Miller H.J., Howkins A.B. (1967). Further analysis of the factors controlling strobilization, differentiation, and maturation of Echinococcus granulosus in vitro. Exp. Parasitol..

[10] Smyth J., Howkins A., Barton M. (1966). Factors controlling the differentiation of the hydatid organism, Echinococcus granulosus, into cystic or strobilar stages in vitro. Nature.

[11] Morseth D.J. (1967). Fine structure of the hydatid cyst and protoscolex of Echinococcus granulosus. J. Parasitol..

[12] Smyth J.D., Gemmell M., Smyth M.M. (1970). Establishment of Echinococcus Granuiosus in the Intestine of Normal and Vaccinated Dogs.

[13] Smyth J.D., Smyth J.D. (1990). Cestoda. In Vitro Cultivation of Parasitic Helminthes.

[14] Parkinson J., Wasmuth J.D., Salinas G., Bizarro C.V., Sanford C., Berriman M., Ferreira H.B., Zaha A., Blaxter M.L., Maizels R.M. (2012). A Transcriptomic Analysis of Echinococcus granulosus Larval Stages: Implications for Parasite Biology and Host Adaptation. PLoS Negl. Trop. Dis..

[15] Tsai I.J., Zarowiecki M., Holroyd N., Garciarrubio A., Sanchez-Flores A., Brooks K.L., Tracey A., Bobes R.J., Fragoso G., Sciutto E. (2013). The genomes of four tapeworm species reveal adaptations to parasitism. Nature.

[16] Zheng H., Zhang W., Zhang L., Zhang Z., Li J., Lu G., Zhu Y., Wang Y., Huang Y., Liu J. (2013). The genome of the hydatid tapeworm Echinococcus granulosus. Nat. Genet..

[17] Huang F., Dang Z., Suzuki Y., Horiuchi T., Yagi K., Kouguchi H., Irie T., Kim K., Oku Y. (2016). Analysis on Gene Expression Profile in Oncospheres and Early Stage Metacestodes from Echinococcus multilocularis. PLoS Negl. Trop. Dis..

[18] Camargo de Lima J., Monteiro K.M., Basika Cabrera T.N., Paludo G.P., Moura H., Barr J.R., Zaha A., Ferreira H.B. (2018). Comparative proteomics of the larval and adult stages of the model cestode parasite Mesocestoides corti. J. Proteom..

[19] Basika T., Paludo G.P., Araujo F.M., Salim A.C., Pais F., Maldonado L., Macchiaroli N., Camargo de Lima J., Rosenzvit M., Oliveira G.C. (2019). Transcriptomic profile of two developmental stages of the cestode parasite Mesocestoides corti. Mol. Biochem. Parasitol..

[20] Basika T., Macchiaroli N., Cucher M., Espínola S., Kamenetzky L., Zaha A., Rosenzvit M., Ferreira H.B. (2016). Identification and profiling of microRNAs in two developmental stages of the model cestode parasite Mesocestoides corti. Mol. Biochem. Parasitol..

[21] Debarba J.A., Monteiro K.M., Moura H., Barr J.R., Ferreira H.B., Zaha A. (2015). Identification of Newly Synthesized Proteins by Echinococcus granulosus Protoscoleces upon Induction of Strobilation. PLoS Negl. Trop. Dis..

[22] Kramer S. (2012). Developmental regulation of gene expression in the absence of transcriptional control: The case of kinetoplastids. Mol. Biochem. Parasitol..

[23] Thorson R.E. (1969). Environmental Stimuli and the Responses of Parasitic Helminths. Bioscience.

[24] Haile S., Papadopoulou B. (2007). Developmental regulation of gene expression in trypanosomatid parasitic protozoa. Curr. Opin. Microbiol..

[25] Thompson R.C.A., Lymbery A.J. (2013). Let’s not forget the thinkers. Trends Parasitol..

[26] Olson P.D., Littlewood D.T.J., Bray R.A., Mariaux J., Olson P.D., Bray R.A., Mariaux J. (2001). Interrelationships and evolution of the tapeworms (Platyhelminthes: Cestoda). Mol. Phylogenet. Evol..

[27] Obal G., Ramos A.L., Silva V., Lima A., Batthyany C., Bessio M.I., Ferreira F., Salinas G., Ferreira A.M. (2012). Characterisation of the Native Lipid Moiety of Echinococcus granulosus Antigen B. PLoS Negl. Trop. Dis..

[28] Pórfido J.L., Herz M., Kiss F., Kamenetzky L., Brehm K., Rosenzvit M.C., Córsico B., Franchini G.R. (2020). Fatty acid-binding proteins in Echinococcus spp.: The family has grown. Parasitol. Res..

[29] Espínola S.M., Ferreira H.B., Zaha A. (2014). Validation of suitable reference genes for expression normalization in Echinococcus spp. larval stages. PLoS ONE.

[30] Mamuti W., Sako Y., Xiao N., Nakaya K., Nakao M., Yamasaki H., Lightowlers M.W., Craig P.S., Ito A. (2006). Echinococcus multilocularis: Developmental stage-specific expression of Antigen B 8-kDa-subunits. Exp. Parasitol..

[31] Roberts A.J., Kon T., Knight P.J., Sutoh K., Burgess S.A. (2013). Functions and mechanics of dynein motor proteins. Nat. Rev. Mol. Cell Biol..

[32] Pao S.S., Paulsen I.T., Saier M.H. (1998). Major facilitator superfamily. Microbiol. Mol. Biol. Rev..

[33] Cantacessi C., Seddon J.M., Miller T.L., Leow C.Y., Thomas L., Mason L., Willis C., Walker G., Loukas A., Gasser R.B. (2013). A genome-wide analysis of annexins from parasitic organisms and their vectors. Sci. Rep..

[34] Siozios S., Ioannidis P., Klasson L., Andersson S.G.E., Braig H.R., Bourtzis K. (2013). The diversity and evolution of Wolbachia ankyrin repeat domain genes. PLoS ONE.

[35] Stankewich M.C., Moeckel G.W., Ji L., Ardito T., Morrow J.S. (2016). Isoforms of Spectrin and Ankyrin Reflect the Functional Topography of the Mouse Kidney. PLoS ONE.

[36] Silva L.L., Marcet-Houben M., Nahum L.A., Zerlotini A., Gabaldón T., Oliveira G. (2012). The Schistosoma mansoni phylome: Using evolutionary genomics to gain insight into a parasite’s biology. BMC Genom..

[37] Hu D., Song X., Xie Y., Zhong X., Wang N., Zheng Y., Gu X., Wang T., Peng X., Yang G. (2015). Molecular insights into a tetraspanin in the hydatid tapeworm Echinococcus granulosus. Parasit. Vectors.

[38] Mousavi S.M., Afgar A., Mohammadi M.A., Mortezaei S., Faridi A., Sadeghi B., Fasihi Harandi M. (2020). Biological and morphological consequences of dsRNA-induced suppression of tetraspanin mRNA in developmental stages of Echinococcus granulosus. Parasit. Vectors.

[39] Cui S.J., Xu L.L., Zhang T., Xu M., Yao J., Fang C.Y., Feng Z., Yang P.Y., Hu W., Liu F. (2013). Proteomic characterization of larval and adult developmental stages in Echinococcus granulosus reveals novel insight into host-parasite interactions. J. Proteom..

[40] Paludo G.P., Thompson C.E., Miyamoto K.N., Guedes R.L.M., Zaha A., Vasconcelos A.T., Sehabiague M.C., Ferreira H.B. Cestode strobilation: Prediction of developmental genes and pathways. BMC Genom.

[41] Zhang R., Aji T., Shao Y., Jiang T., Yang L., Lv W., Chen Y., Chen X., Wen H. (2017). Nanosecond pulsed electric field (nsPEF) disrupts the structure and metabolism of human Echinococcus granulosus protoscolex in vitro with a dose effect. Parasitol. Res..

[42] Balbinotti H., Santos G.B., Badaraco J., Arend A.C., Graichen D.Â.S., Haag K.L., Zaha A. (2012). Echinococcus ortleppi (G5) and Echinococcus granulosus sensu stricto (G1) loads in cattle from Southern Brazil. Vet. Parasitol..

[43] FastQC: A Quality Control Tool for High Throughput Sequence Data. http://www.bioinformatics.babraham.ac.uk/projects/fastqc.

[44] Bolger A.M., Lohse M., Usadel B. (2014). Trimmomatic: A flexible trimmer for Illumina sequence data. Bioinformatics.

[45] Kim D., Pertea G., Trapnell C., Pimentel H., Kelley R., Salzberg S.L. (2013). TopHat2: Accurate alignment of transcriptomes in the presence of insertions, deletions and gene fusions. Genome Biol..

[46] Howe K.L., Bolt B.J., Shafie M., Kersey P., Berriman M. (2017). WormBase ParaSite—A comprehensive resource for helminth genomics. Mol. Biochem. Parasitol..

[47] Feng J., Meyer C.A., Wang Q., Liu J.S., Liu X.S., Zhang Y. (2012). GFOLD: A generalized fold change for ranking differentially expressed genes from RNA-seq data. Bioinformatics.

[48] Wilson D., Pethica R., Zhou Y., Talbot C., Vogel C., Madera M., Chothia C., Gough J. (2009). SUPERFAMILY--sophisticated comparative genomics, data mining, visualization and phylogeny. Nucleic Acids Res..

[49] Jones P., Binns D., Chang H.Y., Fraser M., Li W., McAnulla C., McWilliam H., Maslen J., Mitchell A., Nuka G. (2014). InterProScan 5: Genome-scale protein function classification. Bioinformatics.

[50] Huerta-Cepas J., Szklarczyk D., Forslund K., Cook H., Heller D., Walter M.C., Rattei T., Mende D.R., Sunagawa S., Kuhn M. (2016). eggNOG 4.5: A hierarchical orthology framework with improved functional annotations for eukaryotic, prokaryotic and viral sequences. Nucleic Acids Res..

