# Supplementary material for: Transcriptomic Analysis of the Early Strobilar Development of Echinococcus granulosus
Source: Pathogens. 2020 Jun 12;9(6):465. doi: 10.3390/pathogens9060465 (PMC7350322; doi:10.3390/pathogens9060465)
Supplement: Supplementary file 1 [file pathogens-09-00465-s001.zip › pathogens-816943-supplementary/Supplementary Figure.pdf]

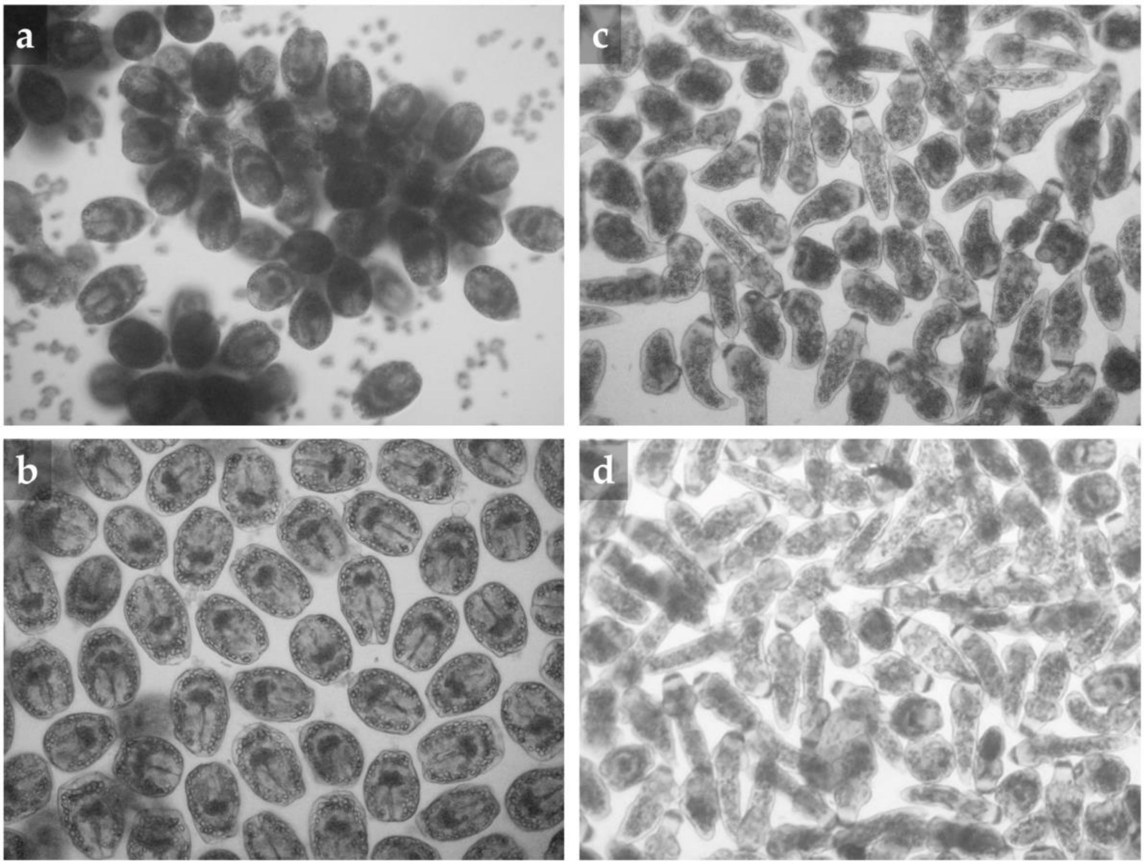

Figure S1. Induction of strobilar development in *E. granulosus* protoscoleces. (a) Protoscoleces washed with phosphate-buffered saline and (b) after pepsin treatment. (c) Protoscoleces after 12 and (d) 24 hours in complete biphasic medium. Magnification 100x.
