# Supplementary material for: Transcriptomic Analysis of the Early Strobilar Development of Echinococcus granulosus
Source: Pathogens. 2020 Jun 12;9(6):465. doi: 10.3390/pathogens9060465 (PMC7350322; doi:10.3390/pathogens9060465)
Supplement: Supplementary file 1 [file pathogens-09-00465-s001.zip › pathogens-816943-supplementary/Supplementary tables/Table S3.docx]

**Table S3.** Selected differentially expressed genes among the samples analyzed of *E. granulosus.*

|  |  | **RPKM** | | | | **Profile** | | | |  |  |
| --- | --- | --- | --- | --- | --- | --- | --- | --- | --- | --- | --- |
| **Gene_ID** | **Gene_Name** | **PBS** | **PEP** | **12 h** | **24 h** | **PBSx12 h** | **PBSx24 h** | **PEPx12 h** | **PEPx24 h** | **Superfamily^1^** | **EggNOG^2^** |
| EgrG_000107200 | 2 amino 3 ketobutyrate coenzyme A ligase | 201,774 | 203,383 | 1583,37 | 1074,42 | Up | Up | Up | Up | RB | E |
| EgrG_000117000 | Ankyrin | 24,0945 | 17,9373 | 59,5489 | 24,153 | - | - | Up | - | RD | O |
| EgrG_001165500 | Ankyrin | 74,7373 | 67,9019 | 246,727 | 182,952 | Up | Up | Up | Up | RD | E |
| EgrG_000193700 | Annexin | 1095,57 | 1303,16 | 443,858 | 833,11 | Down | - | Down | - | IA | U |
| EgrG_000244000 | Annexin | 872,956 | 973,713 | 303,621 | 434,858 | Down | - | Down | Down | IA | U |
| EgrG_000330300 | Annexin | 953,654 | 973,326 | 467,045 | 427,206 | Down | Down | Down | Down | IA | U |
| EgrG_000124900 | Aquaporin 4 | 195,726 | 234,991 | 28,6044 | 41,8997 | Down | Down | Down | Down | RF | G |
| EgrG_000153200 | Aquaporin 9 AQP 9 small solute channel 1 | 6,07709 | 6,86419 | 325,38 | 156,091 | Up | Up | Up | Up | RF | G |
| EgrG_001190800 | Aquaporin 9 AQP 9 small solute channel 1 | 11,3987 | 8,44232 | 422,027 | 210,038 | Up | Up | Up | Up | RF | G |
| EgrG_000203700 | Choline transporter protein 2 | 72,4286 | 75,4026 | 30,3665 | 33,5379 | Down | - | Down | Down | - | P |
| EgrG_000127200 | Concentrative Na nucleoside cotransporter | 0,472568 | 1,20989 | 287,929 | 113,074 | Up | Up | Up | Up | - | P |
| EgrG_000342900 | Cysticercus cellulosae specific antigenic | 18289,6 | 20966,8 | 117,226 | 204,453 | Down | Down | Down | Down | - | - |
| EgrG_000261100 | Diagnostic antigen gp50 | 9,18014 | 6,89773 | 1,77943 | 2,37523 | Down | - | Down | - | - | - |
| EgrG_000564000 | Diagnostic antigen gp50 | 441,138 | 466,854 | 46,5439 | 81,7405 | Down | Down | Down | Down | - | - |
| EgrG_000520550 | Diagnostic antigen gp50 | 0,446094 | 0 | 8,28655 | 3,982 | Up | Up | Up | Up | - | - |
| EgrG_000304800 | Diagnostic antigen gp50 | 3,21728 | 2,40246 | 161,229 | 133,223 | Up | Up | Up | Up | - | - |
| EgrG_000324200 | Diagnostic antigen gp50 | 104,485 | 115,282 | 568,807 | 422,461 | Up | Up | Up | Up | - | - |
| EgrG_000566700 | Diagnostic antigen gp50 | 145,332 | 149,558 | 497,9 | 427,249 | Up | Up | Up | Up | - | - |
| EgrG_000071400 | Dynein light chain | 574,633 | 776,583 | 374,921 | 400,581 | - | - | Down | - | N | Z |
| EgrG_000941000 | Dynein light chain | 1522,5 | 2014,49 | 431,851 | 361,5 | Down | Down | Down | Down | N | Z |
| EgrG_000941100 | Dynein light chain | 2298,07 | 3266,25 | 512,348 | 697,2 | Down | Down | Down | Down | N | Z |
| EgrG_000940900 | Dynein light chain | 3620,31 | 4518,12 | 1492,48 | 1444,34 | Down | Down | Down | Down | N | Z |
| EgrG_000347100 | Elongation of very long chain fatty acids | 168,956 | 193,03 | 429,035 | 277,86 | Up | - | - | - | - | I |
| EgrG_000321300 | Elongation of very long chain fatty acids | 145,857 | 168,369 | 379,318 | 343,661 | Up | - | - | - | - | I |
| EgrG_000549800 | Fatty acid binding protein FABP2 | 1195,61 | 1125,07 | 497,465 | 454,409 | Down | Down | Down | Down | RF | I |
| EgrG_000549850 | Fatty acid binding protein FABP2 | 15557,9 | 17680,6 | 7030,25 | 5519,12 | Down | Down | Down | Down | RF | I |
| EgrG_000500800 | Forkhead box protein J3 | 6,74812 | 10,582 | 25,906 | 19,0748 | Up | - | - | - | LA | K |
| EgrG_000159700 | Forkhead box protein K1 | 56,2761 | 55,7012 | 16,4889 | 13,4864 | Down | Down | Down | Down | LA | K |
| EgrG_000179000 | Forkhead box protein O4 | 127,353 | 101,933 | 47,5055 | 44,8686 | Down | Down | Down | Down | LA | K |
| EgrG_000320300 | Forkhead box protein P4 | 84,3874 | 94,8522 | 42,8213 | 33,9381 | - | Down | Down | Down | LA | K |
| EgrG_000166300 | Forkhead box Q:D protein transcription | 57,4808 | 55,836 | 168,579 | 133,053 | Up | - | Up | - | LA | K |
| EgrG_000521000 | Heat shock protein 70 | 10,0821 | 6,12389 | 69,4092 | 45,7634 | Up | Up | Up | Up | RC | O |
| EgrG_000520950 | Heat shock protein 70 | 6,45234 | 6,57028 | 108,38 | 50,6147 | Up | Up | Up | Up | RC | O |
| EgrG_000554100 | Heat shock protein 70 | 37,0527 | 31,2413 | 324,553 | 280,569 | Up | Up | Up | Up | O | O |
| EgrG_000772900 | Homeobox protein ceh 26 | 9,33366 | 9,52123 | 2,16725 | 6,50904 | Down | - | Down | - | LA | K |
| EgrG_000040400 | Krueppel factor 10 | 559,331 | 589,885 | 127,123 | 114,181 | Down | Down | Down | Down | LA | K |
| EgrG_000608700 | Krueppel factor 5 | 317,941 | 424,699 | 35,579 | 28,4103 | Down | Down | Down | Down | LA | K |
| EgrG_000419100 | Kunitz protease inhibitor | 1520,32 | 1792,19 | 769,481 | 648,642 | - | Down | Down | Down | OA | - |
| EgrG_000445600 | Long chain fatty acid coenzyme A ligase 5 | 395,132 | 314,496 | 137,806 | 124,926 | Down | Down | Down | Down | RC | I |
| EgrG_000212700 | Major egg antigen p40 | 2923,9 | 3139,22 | 1186,33 | 848,683 | Down | Down | Down | Down | O | O |
| EgrG_000586900 | Oxalate:formate antiporter | 58,5919 | 46,314 | 22,5862 | 26,3801 | Down | - | - | - | P | G |
| EgrG_000586800 | Oxalate:formate antiporter | 9,41304 | 6,93566 | 56,9295 | 24,2628 | Up | - | Up | Up | P | G |
| EgrG_000017400 | Oxalate:formate antiporter | 186,078 | 190,454 | 28,2336 | 36,1629 | Down | Down | Down | Down | P | G |
| EgrG_000661800 | Oxalate:formate antiporter | 248,386 | 231,971 | 33,6004 | 48,2633 | Down | Down | Down | Down | P | G |
| EgrG_000204100 | P53 transcription factor DNA binding | 98,8238 | 81,228 | 39,9723 | 41,4273 | Down | Down | - | - | - | - |
| EgrG_000249100 | Phospholipase d1 | 0,193565 | 0,27876 | 19,9917 | 17,2063 | Up | Up | Up | Up | RC | I |
| EgrG_000204600 | Potassium voltage gated channel subfamily D | 6,35751 | 4,88303 | 0,944766 | 3,31039 | Down | - | - | - | P | P |
| EgrG_000197100 | Proto oncogene serine:threonine protein kinase | 147,414 | 116,318 | 48,2426 | 38,11 | Down | Down | Down | Down | OB | T |
| EgrG_001001900 | Pyruvate kinase | 0,569787 | 2,55289 | 5,50381 | 3,67332 | Up | Up | - | - | C | G |
| EgrG_001127400 | Sodium bile acid cotransporter | 0,767959 | 0,552983 | 5,13557 | 2,85629 | Up | - | Up | - | - | - |
| EgrG_001127500 | Sodium bile acid cotransporter | 13,8243 | 13,0956 | 76,5597 | 56,7643 | Up | Up | Up | Up | - | P |
| EgrG_000381100 | Tapeworm specific antigen B | 18,6673 | 6,72087 | 2,6007 | 1,73574 | Down | Down | - | - | - | - |
| EgrG_000381600 | Tapeworm specific antigen B | 59,6463 | 93,9159 | 252,619 | 231,605 | Up | Up | - | - | - | - |
| EgrG_000381400 | Tapeworm specific antigen B | 705,858 | 551,111 | 25,1401 | 58,1474 | Down | Down | Down | Down | - | - |
| EgrG_000381200 | Tapeworm specific antigen B | 2680,18 | 2204,6 | 171,536 | 310,395 | Down | Down | Down | Down | - | - |
| EgrG_000381700 | Tapeworm specific antigen B | 34,8619 | 42,5989 | 167,195 | 157,953 | Up | Up | Up | Up | - | - |
| EgrG_000381800 | Tapeworm specific antigen B | 140,34 | 217,926 | 2490,87 | 3299,76 | Up | Up | Up | Up | - | - |
| EgrG_000328400 | Tetraspanin | 35,7626 | 42,9192 | 59,7888 | 105,163 | - | Up | - | - | RE | S |
| EgrG_001077400 | Tetraspanin | 131,894 | 137,408 | 308,601 | 578,232 | - | Up | - | Up | RE | S |
| EgrG_000873600 | Universal stress protein | 2596,12 | 3003,99 | 501,033 | 436,72 | Down | Down | Down | Down | F | T |
| EgrG_000811100 | Universal stress protein | 13,3471 | 25,0464 | 224,943 | 152,537 | Up | Up | Up | Up | F | T |
| EgrG_001017500 | Zinc finger protein | 50,7582 | 65,6282 | 17,2905 | 17,7426 | Down | Down | Down | Down | LA | K |
| EgrG_000186000 | Zinc finger protein 45 | 13,5889 | 10,3948 | 1,88806 | 2,05454 | Down | Down | Down | Down | LA | K |
| EgrG_000711000 | Zinc finger transcription factor gli2 | 35,5351 | 41,3339 | 14,4482 | 11,1371 | Down | Down | Down | Down | LA | K |
| EgrG_000737300 | Zinc transporter foi | 18,7469 | 24,4141 | 59,7177 | 72,487 | Up | Up | - | Up | - | P |

(-) Not differentially expressed

^1^Superfamily codes: C – energy; F - nucleotide metabolism and transport; IA – phospholipid metabolism and transport; LA – DNA-binding; N – cell motility; O – protein modification; OA – proteases; OB - kinases/phosphatases; P – ion metabolism and transport; RB – transferases; RC – other enzymes; RD – protein interaction; RE - immune response; RF – transport.

^2^EggNOG functional categories: E – amino acid transport and metabolism; G – carbohydrate transport and metabolism; I – lipid transport and metabolism; K – transcription; O – post-translational modification, protein turnover, and chaperones; P – inorganic ion transport and metabolism; S – function unknown; T – signal transduction mechanisms; U – intracellular trafficking, secretion, and vesicular transport; Z – cytoskeleton.
